# Assessing the benefits and usefulness of Schwartz Centre rounds in second-year medical students using clinical educator-facilitated group work session: not just “a facilitated moan”!

**DOI:** 10.1186/s12909-020-02199-x

**Published:** 2020-08-17

**Authors:** J. Smith, M. G. Stewart, E. Foggin, S. Mathews, J. Harris, P. Thomas, A. Cooney, C. J. Stocker

**Affiliations:** Medical School, University of Buckingham, Hunter Street, Buckingham, MK18 1EG UK

**Keywords:** SR, Undergraduate medical education, Resilience, Compassion, Empathy

## Abstract

**Background:**

An experiential curriculum exposing medical students to the clinic early has many benefits but comes with the emotional stress this environment engenders. Schwartz rounds (SR) are an effective means to combat emotional stress and increasingly used in UK and USA hospitals. Recent studies show that the SR format may also provide benefits for medical students. This study aimed to investigate whether the guidance of SR in second year medical students provides the same benefits as to healthcare professionals.

**Methods:**

SR assessment involved 83 s year MBChB students in facilitated groupwork sessions. Topics discussed were “change and resilience” and “duty of candour”. Students completed a Likert Scale questionnaire evaluating outcomes proffered by the Point of Care Foundation in collaboration with the Schwartz Foundation, with freeform feedback.

**Results:**

There was an 86% completion rate with 25% providing written feedback. Participants were more likely to agree than disagree that SR were beneficial. SR effectiveness in enhancing students’ working relationship awareness and skills was strongly correlated with understanding the purpose of, and engagement with, the SR (*P* < 0.001). Similarly, engagement with the SR was strongly correlated with self-reporting of enhanced patient-centredness (*P* < 0.001). Freeform feedback could be grouped into five themes that revolved around understanding of the SR and engagement with the process. Many positive comments regarded the SR as a forum not only to “learn experientially” but to so in a “safe environment”. Many negative comments stemmed from students not seeing any benefits of engagement with the SR, in that sharing experiences was “unbeneficial”, “empathy is inherent and not learnt”, or that sharing emotional problems is simply “moaning”.

**Conclusion:**

SRs are an effective way of fostering empathy and understanding towards patients and colleagues. However, for the students to benefit fully from the SR it is necessary for them to engage and understand the process. Therefore, for the successful implementation of SR into pre-clinical medical education, it is important to help students realise that SR are not merely a “facilitated whinge”.

## Background

With the introduction of spiral curricula, medical education has evolved such that more medical students are being exposed to clinical environments at earlier stages in their training [1]. Although it offers an important role in the contextualisation of theory, it is important to remember that this high-pressure environment can result in increased levels of stress and emotional unrest, all of which can affect one’s ability to maintain excellent standards of patient care [2, 3]. What is more, when students graduate, many feel ill-equipped to deal with issues such as managing upset relatives, breaking bad news, and resolving conflict with co-workers [4]. Medical schools have a responsibility to prepare their students for the workplace by developing their resilience to stressful situations [5]. The General Medical Council (GMC) [6] recognises this and states that students should have insight into their own mental health and “develop healthy ways to cope with stress and challenges” [7]. It should therefore be considered how medical schools can best facilitate and promote effective coping mechanisms at the earliest opportunity, particularly those that expose students to the clinical environment in the initial stages. Two domains that are likely to contribute to resilience are emotional intelligence and the ability to reflect [8].

One initiative that has allowed caregivers to share and reflect on these challenging clinical experiences is the Schwartz Round (SR), formulated and trademarked by the Schwartz Center for Compassionate Healthcare, Boston, USA. The aim of the SR is to help healthcare workers cope with the stress of providing compassionate care and the emotional drain that often accompanies this [8]. Although implementation varies, SRs offer a unique form of support and can improve well-being and increase empathy towards patients and colleagues. SRs are unlike Grand Rounds, Balint Groups and Debriefings as they are open to all staff, including those non-clinical, and topics are used as a springboard for a wider discussion.

Since 2009, SRs have rapidly spread across UK hospitals with attendees reporting it was useful to learn how others have dealt with similar challenging scenarios and have become more empathic and respectful towards colleagues and patients [9–11]. Though initially designed for hospital staff, SRs have been piloted with Year 5 and 6 medical students, with the majority agreeing that it was a useful tool giving insight into others’ views [12]. Results have been similarly encouraging when looking at incorporating SRs into earlier stages of training, specifically second-year undergraduates with limited clinical exposure. However, this resulted in some respondents feeling as though their inexperience reduced the effectiveness of the exercise [13].

If effective, SRs may have the potential to be used as an educational tool to enhance reflective skills to better prepare students for their future careers as doctors. Questions remain as to whether SRs could effectively be incorporated into undergraduate medical curricula and if so, how they might be adapted to enhance the experience for early-year students who have limited clinical exposure. This study allowed learners to discuss both non-clinical and clinical scenarios in an SR facilitated by ‘Clinical Educators’ (CEs), that is junior doctors with an interest in medical education, most of whom have just completed their Foundation Year 2 training. CEs facilitate interactive group work sessions as part of the curriculum and provide ‘near-peer’ support to students, which has been demonstrated to enhance learning of skills [14] and patient-centredness [15].

This study aims to explore whether the additional guidance and experiences of these junior doctor role models enriches students’ understanding and appreciation of the SR by providing a realistic vision of where the learners will be in several years. It has been shown that students “want to hear from ‘real’ professionals, not archetypes” [16] and in doing so can better develop coping strategies.

## Methods

### Thick description of transferability: research design, Programme description and setting

This study comprised a single session and was piloted with the entire cohort of 83 s-year medical students on a single MBChB programme at the University of Buckingham. Initiating the session was a 20-min lecture with an introduction to SRs delivered by a trained facilitator. The cohort was then evenly divided into two identical group work rooms. It was a familiar and neutral environment and the room was arranged to ensure there were no physical barriers or interruptions. The students were seated in a semicircle around a panel composed of one consultant and two CEs. The first theme was introduced:

### Change and resilience: think about the difficulty in coming to a new healthcare environment and how you adapted. How did you feel introducing yourselves to patients, examining patients, considering your and their vulnerability?

Panel members opened with a discussion of the theme before sharing relevant personal experiences for 10 min. Facilitated discussion among students continued for a further 40 min. The second topic followed the same format and timings:

### Duty of candour: think about any adverse incidences, clinical or non-clinical, you have seen. Consider the safety implications to patients and colleagues

A short de-brief and closing statement concluded the session.

The entire cohort of second-year medical students on the MBChB programme were included. Attendance was monitored. The group consisted of 36 male and 47 female, 75 single, 7 married and 1 divorced. Ages ranged from 18 to over 40 (average age 24 ± 6 standard deviation). The percentage of international students was 42%. Throughout Year 1 and Year 2, students spend a half-day every week developing their clinical skills: these sessions are equally divided into primary care, secondary care and on-site at the university. During these themed sessions, students practise history-taking and examination with patients. In hospitals, students may be allocated to a general medical or surgical ward, or a ward specific to the system they are learning about, such as a respiratory ward. Ward-based teaching may be delivered by consultants or junior doctors.

### Data collection and analysis

The students were given a feedback form immediately after the SR, asking them to evaluate the Point of Care Foundation (POCF) outcomes [17] using a 5-part Likert scale (completely disagree to completely agree), and to supply in white spaces how they thought the SR had impacted on specific aspects of their professional identity. The Point of Care Foundation is a UK-based non-for-profit organisation with a mission to humanise healthcare. One of their roles is the facilitation of SR implementation across different organisations). Likert scale responses were analysed descriptively by frequency, mode and median. Statistical correlations were analysed with Spearman’s rank correlation analysis. The qualitative data was coded by two authors (a senior lecturer and a junior doctor) and a brief qualitative thematic analysis was performed; the Kappa coefficient for the inter-rater reliability of the coding was 0.84. Data was anonymized by a senior faculty member. Statistical analyses were carried out using IBM Statistical Package for the Social Sciences (SPSS) version 20.

The ten statements the students were asked to score were:

1. Today’s Round will help me work better with my colleagues.
2. In today’s Round I have gained knowledge that will help me care for patients.
3. Today’s Round has given me confidence in handling non-clinical aspects of care.
4. Today’s Round has given me greater awareness in handling sensitive issues.
5. Today’s Round has me greater understanding of how expressing thoughts, questions and feelings would help me.
6. Today’s Round has given me greater understanding of how giving and receiving support is beneficial and helps us feel valued.
7. Today’s Round has given me a greater awareness of improving teamwork, connectness and communication.
8. Today’s Round has given me greater awareness of the importance of attentiveness to social and emotional aspects of patient care.
9. Today’s Round has given me an awareness of increased feelings of compassion towards patients.
10. Today’s Round has given me a greater understanding of the importance of empathy with patients as people.

### Rigor

Quality of the written response data was ensured by the following methods: analyst triangulation was used to ensure data credibility with two analysts from different backgrounds (a senior lecturer and a junior doctor) independently coding the thematic analysis; a thick description of the study (see above); dependability and confirmability was ensured by an external audit by a researcher outside the Medical School, from the Institute of Translational Medicine, Buckingham, UK.

The SR was implemented according to POCF guidance [17]. The POCF is a UK-based non-for-profit organisation with a mission to humanise healthcare. One of their roles is to ensure the standardisation of SR implementation across different organisations. They provide guidance on how to introduce the SR, choosing a suitable venue, the room layout, the timing of SRs, and selecting topics. The official POCF feedback template was implemented to gather students’ responses. The same set of Likert scale questions is asked of all Rounds’ participants in the UK and US and is standardized and is part of the POCF licence agreement with the Schwartz Center for Compassionate Healthcare™ to ensure validity, reliability and reproducibility of the study. This study has been approved by the University of Buckingham Science and Medicine Ethical Review panel.

## Results

### Students’ performance in the SR

Of the 83 students in the cohort, 82 returned the questionnaire following the SR; 71 answered all questions and 21 gave written feedback. This represents an 86% completion rate with 25% providing written feedback. Responses to the POCF outcomes questionnaire are shown in Table 1. For each of the ten outcomes there was a greater number of positive (completely or somewhat agree) returns than negative (completely or somewhat disagree) returns.

**Table 1:** Responses to the Point of Care Foundation (POCF) outcomes using a 5-part Likert scale. Frequencies are shown for each Likert item with the mode and median item. Questions 1–10 are: Q1. Today’s Round will help me work better with my colleagues; Q2. In today’s Round I have gained knowledge that will help me care for patients; Q3.Today’s Round has given me confidence in handling non-clinical aspects of care; Q4. Today’s Round has given me greater awareness in handling sensitive issues; Q5. Today’s Round has me greater understanding of how expressing thoughts, questions and feelings would help me; Q6. Today’s Round has given me greater understanding of how giving and receiving support is beneficial and helps us feel valued; Q7. Today’s Round has given me a greater awareness of improving teamwork, connectness and communication; Q8. Today’s Round has given me greater awareness of the importance of attentiveness to social and emotional aspects of patient care; Q9. Today’s Round has given me an awareness of increased feelings of compassion towards patients; Q10. Today’s Round has given me a greater understanding of the importance of empathy with patients as people

Statement	Number of students					Mode score	Median score
	Completely disagree	Somewhat disagree	Neither agree nor disagree	Somewhat agree	Completely agree		
Q1	1	5	24	29	19	Somewhat agree	Somewhat agree
Q2	1	6	15	31	25	Somewhat agree	Somewhat agree
Q3	1	4	16	32	25	Somewhat agree	Somewhat agree
Q4	2	2	10	36	28	Somewhat agree	Somewhat agree
Q5	0	1	14	32	20	Somewhat agree	Somewhat agree
Q6	0	2	13	33	19	Somewhat agree	Somewhat agree
Q7	0	6	13	29	19	Somewhat agree	Somewhat agree
Q8	0	3	12	32	20	Somewhat agree	Somewhat agree
Q9	0	6	15	29	19	Somewhat agree	Somewhat agree
Q10	1	2	15	24	25	Completely agree	Completely agree

Written commentary could be grouped into the following five themes:

- Professionalism as a binary entity
- Unable to relate
- Attention-seeking and an opportunity to complain
- Sharing and empathising in a safe space
- Learning from others’ experiences

Most students gave positive feedback about the perceived benefits of the rounds, with no students responding negatively in all 5 themes.

### Students who gain understanding and engagement with the SR are more likely to reap the benefits of the round regarding working relationship skills and enhanced patient-centredness

The success of raising understanding of and engagement with the SR was addressed by the three criteria: (i) “Q5. greater understanding of how expressing thoughts, expressions and feelings can help me”; (ii) Q6. giving and receiving support is beneficial and helps us all”; and (iii) Q8. greater awareness of the importance of attentiveness to social and emotional aspects of patient care”. Spearman correlation coefficient analysis shows a strong positive correlation (*P* < 0.0001) between the responses to each of these criteria and the responses to questions designed to analyse SR effectiveness in enhancing students’ working relationship awareness and skills (Table 2). These were: (i) “Q1. work better with my colleagues”; (ii) “Q3. gained confidence in handling non-clinical aspects of care”; and (iii) “Q7. greater awareness of improving teamwork, connectedness and communication. Likewise, the students whose experience of the SR raised their understanding and engagement also had a strong positive correlation with those self-reporting an enhanced patient-centredness (Table. 3). This criterion was assessed with the following four questions: (i) “Q2. gained knowledge that will help care for patients”; (ii) “Q4. greater awareness in handling sensitive issues”; (iii) “Q9. awareness of increased feelings of compassion towards patients”; (iv) “Q10. greater understanding of the importance of empathy with patients as people”.

**Table 2:** There is a strong statistically significant correlation between students who obtained a) “Q5. greater understanding of how expressing thoughts, expressions and feeling can help me”; b) “Q6. have greater understanding of how giving and receiving support is beneficial”; and c) “Q8. greater awareness of the importance of attentiveness to social and emotional aspects of patient care”, are the students who enhanced their working relationship awareness and skills (Q1, Q3 and Q7). Correlation analyses are by Spearman’s rank correlation test with vales for rho (*ρ*) and statistical significance (*P*) shown

	Q1 Today’s Round will help me work better with my colleagues	Q3. Today’s Round has given me confidence in handling non-clinical aspects of care	Q7. Today’s Round has given me a greater awareness of improving teamwork, connectness and communication
Q5. Today’s Round has me greater understanding of how expressing thoughts, questions and feelings would help me	*ρ* = 0.49 *P* < 0.0001	*ρ* = 0.68 *P* < 0.0001	*ρ* = 0.54 *P* < 0.0001
Q6. Today’s Round has given me greater understanding of how giving and receiving support is beneficial and helps us feel valued	*ρ* = 0.55 *P* < 0.0001	*ρ* = 0.68 *P* < 0.0001	*ρ* = 0.65 *P* < 0.001
Q8. Today’s Round has given me greater awareness of the importance of attentiveness to social and emotional aspects of patient care	*ρ* = 0.50 *P* < 0.0001	*ρ* = 0.59 *P* < 0.0001	*ρ* = 0.59 *P* < 0.001

**Table 3:** There is a strong statistically significant correlation between students who obtained a) “Q5. greater understanding of how expressing thoughts, expressions and feeling can help me”; b) “Q6. have greater understanding of how giving and receiving support is beneficial”; and c) “Q8. greater awareness of the importance of attentiveness to social and emotional aspects of patient care”, and their patient-centredness (Q2, Q4, Q9 and Q10). Correlation analyses are by Spearman’s rank correlation test with vales for rho (*ρ*) and statistical significance (*P*) shown

	Q2. In today’s Round I have gained knowledge that will help me care for patients.	Q4. Today’s Round has given me greater awareness in handling sensitive issues.	Q9. Today’s Round has given me an awareness of increased feelings of compassion towards patients.	Q10. Today’s Round has given me a greater understanding of the importance of empathy with patients as people.
Q5. Today’s Round has me greater understanding of how expressing thoughts, questions and feelings would help me	*ρ* = 0.68 *P* < 0.0001	*ρ* = 0.72 *P* < 0.0001	*ρ* = 0.66 *P* < 0.0001	*ρ* = 0.63 *P* < 0.0001
Q6. Today’s Round has given me greater understanding of how giving and receiving support is beneficial and helps us feel valued	*ρ* = 0.68 *P* < 0.0001	*ρ* = 0.61 *P* < 0.0001	*ρ* = 0.63 *P* < 0.001	*ρ* = 0.68 *P* < 0.001
Q8. Today’s Round has given me greater awareness of the importance of attentiveness to social and emotional aspects of patient care	*ρ* = 0.59 *P* < 0.0001	*ρ* = 0.58 *P* < 0.0001	*ρ* = 0.54 *P* < 0.001	*ρ* = 0.61 *P* < 0.001

### Attitudes to SR based on responses to questionnaires

Of the 21 students who provided written feedback in the questionnaires, the following 5 themes were identified:

1. *Professionalism as a binary entity*

Themes from the written feedback suggested that some students felt that professionalism, compassion and empathy were either qualities that you ‘have’ or ‘don’t have,’ suggesting they do not consider them values that can be developed or improved upon. The students commented that: “*I hope that I already am professional, understanding and tolerant*” (student 10), we “*should already feel compassion*” (student 18) and “*those who have the insight to know they have been affected emotionally by a situation will already seek help and advice from people they trust or those professionally employed to assist them*.” (student 8).

2. *Inability to relate*

A few students appeared to find it difficult to see the value in sharing experiences, seeming to understand the purpose of the SR but perhaps not the relevance to them, or what to do with this information; “*I just feel like they were telling stories from their time in hospital but I did not see how that would affect how I act around my colleagues*” (student 2), I “*gained understanding, but didn’t find it completely effective*” (student 6) and the “*stories were very vague, not very to the point*” (student 13).

3. *Attention-seeking and an opportunity to complain*

Two students found some views particularly difficult to connect with, describing attention-seeking behaviours amongst their peers and suggesting that the SRs are a platform to promote a culture of complaining. They noted: “*people wanted to talk about themselves and it sort of turned into a complaints session … people just try to come up with more extreme stories and how they were victimised*” (student 10) or “*Forced group reflection is just another opportunity for those who are unlikely to have self-insight, or self-aggrandisement from telling their side of the story. Facilitated whinging session*” (student 8).

4. *Sharing and empathising in a safe space*

Conversely, several students described the SR as promoting shared empathy, providing a space to explore emotions safely and express thoughts, questions and feelings; “*I have learned to empathise better with my colleagues … I learned what might go on in other peoples’ minds*” (student 3) and “*understand the usefulness of the rounds and the importance of speaking up*” (student 1), ‘*understood that others feel similar to me*” (student 12) and “*I would feel more confident to speak up about how I feel*” (student 5).

5. *Learning from others’ experiences*

Many students reflected on the SR as a tool to hear colleagues’ experiences and how they dealt with sensitive issues. One student stated that “*hearing others’ experiences has prepared me for potentially difficult situations*” (student 14) and another learned “*how to deal with adverse reactions and situations and about duty of candour*” (student 12). Two students directly commented on the input of CEs mentioning that “*Clinical educators had useful past experiences*” (student 15) and that “*advice was given to guide us in approaching different situations which was somewhat useful*” (student 1). Students felt it was “*useful to share others’ experiences*” (student 16) and “*learnt stories from colleagues*” (student 19) including “How to handle racism and inappropriate comments (student 12). One student “learnt emotional regulation techniques” (student 3).

## Discussion

### Positive outcomes

Overall, the results of the study suggest that the SR was a constructive experience for the students. Most reported that the SR would have a positive impact on their patient care and relationships with colleagues through empathising with and appreciating their colleagues’ perceptions. Approximately 73% of students agreed that the SR enabled a greater understanding of the importance of empathy with patients. This is a similar proportion to the 80% of Year 5 and Year 6 students who found SR enhanced their patient-centredness [12]. They also describe a growth in confidence when it comes to handling non-clinical aspects of care, sensitive issues and challenging scenarios through learning from others’ experiences in the SR. Student 14 stated that “hearing others’ experiences has prepared me for potentially difficult situations” and for student 12 it taught them “how to deal with adverse reactions and situations”. Listening to others promoted a greater awareness of how to improve teamwork and connectedness. These aspects of SR have not been investigated before in medical students, although a preliminary study did find an enhanced awareness of nonclinical, social and emotional aspects of caring for patients in hospital staff [10].

Following the SR, most students agreed that they had a better understanding of how expressing thoughts and feelings could help them, and that giving and receiving support is beneficial to helping them to feel valued. One commented that the SR highlighted the “importance of speaking up” (student 1). Those that understood the pertinence of expressing one’s thoughts and emotions were significantly more likely to benefit from the SR in a variety of ways. Not only were they more likely to come away from the SR realising the importance of attentiveness to social and emotional aspects of patient care, but also were the ones who enhanced their working relationship awareness and skills and their patient-centredness. This is the first study in either students or healthcare professionals that demonstrates that engagement with the SR is key to gaining the advantages. Most other papers report a high level of feedback, which may indicate a natural willingness to engage in SR. However, it may be worth considering the question of how to improve engagement when scheduling SR in medical education.

### The role of clinical educators

The GMC [5] indicates that students “gain coping strategies by talking to their peers and from clinicians who are brought in to talk about real-life experiences [and] who have made mistakes”. Studies on such ‘near-peer’ session facilitation support the GMC stance [14, 15]. Moreover, it has been shown that students “want to hear from ‘real’ professionals, not archetypes” and in doing so can better develop coping strategies [16]. Although the impact of CEs was not directly measured in this study, there is indirect evidence from the feedback to suggest their inclusion was beneficial to the SR. Further investigations into SR efficacy in pre-clinical medical education may benefit from considering the professional identity and/or role of the facilitators in the learning environment.

### Critical feedback

Some students implied the SR was less relevant to them because they “already [felt] compassion” (student 18) and are “already [ …] professional, understanding and tolerant” (student 10). It could be argued that these compassionate role models should utilise the SR to support their peers who may benefit from an open discussion. In doing so, they may well gain something from the SR themselves as was demonstrated by those students who did engage. It may be that these students are less self-aware of their empathy skills. Student 10, who already feels compassionate, also likened the SR to a “complaints session” with “people just try [ing] to come up with more extreme stories of how they were victimised” (student 10). This feedback contradicts the student’s self-description and may demonstrate a lack of insight or understanding of how to maintain high levels of empathy through an exercise such as this. These students may be in danger of entrapment within a self-propagating negative cycle of “lack of awareness” leading to “non-engagement” leading to “non-beneficial Round” – leading to “no enhancement of awareness” and so on. Further work may be needed to improve SR engagement as it may be that the students who would benefit the most from SR are the ones most in danger of receiving no benefit.

Using the SR as a platform to complain was also identified by student 8 who felt that “forced group reflection is just another opportunity for those who are unlikely to have self-insight, or self-aggrandisement from telling their side of the story”. This student has perhaps not fully understood the purpose of an SR. It should be noted that SRs are not primarily intended to be Communities of Practice that spread skills but rather a platform to alleviate the emotional stress that comes with being a healthcare professional, which is achieved through participants sharing their version of events. Consequently, students who are described as complaining are voicing their emotions and using the SR as intended. It is then up to participants to seek a resolution or make sense of the emotions because everyone is valued equally. Therefore, it could be concluded that the purpose of the SR could perhaps be better explained to students beforehand in their briefing. It should also be explored how attitudes to compassion and empathy may be addressed to promote a more understanding environment.

### Limitations

The response rate for the written feedback is relatively low, which may introduce nonresponse, sampling or selection bias. Similarly, the SR was not repeated, and the feedback forms were anonymized. These factors limit generalizability of the findings and analysis of confounding demographic effects. The Likert Scale is a powerful and commonly-used bipolar rating system, but is not specifically designed to rate empathy, resilience, or professional identity. Future studies would ideally use a Scale that does this, e.g. Professional Self Identity Questionnaire, Jefferson Empathy Scale, Resilience Questionnaire, COPE Inventory, or Emotional Intelligence Questionnaire. Third, self-reported data may be vulnerable to social desirability bias.

### Take-home messages

From this study, the authors feel that it is feasible to incorporate SRs into early undergraduate medical education. From the results, it is evident that most students feel that SRs will improve their patient care, teamwork, and communication. There is a role for CEs in acting as an imperfect role model and providing a pertinence to the exercise. The results show that early-year undergraduate medical student generally engage positively with SRs and demonstrate an ability to empathise with each other and share feelings regarding early clinical exposure without inhibition. However, some students find SRs less helpful and feel their peers use it as a platform to complain. The correlation analyses suggest that the students who engage with the SR and gain an understanding of its purpose are also the students who gain the most awareness of the emotional needs of themselves, their colleagues, and patients. Further research on self-rated compassion in early-year students along with the barriers to engagement may be useful, such as demographics which have previously been found to affect SR effectiveness in healthcare professionals [18].

## Conclusions

SRs are an effective way of fostering empathy and understanding towards patients and colleagues in the healthcare environment. When piloted among second-year medical students, though some felt their colleagues were exploiting the exercise to complain, most students felt it would improve their patient care, teamwork and communication skills. There is some indirect evidence that the inclusion of CEs made the SR feel more pertinent to the students with the junior doctors’ clinical experiences being described as a useful addition to the discussion. Suggestions for future research include assessing students’ self-perceived empathy skills and whether they feel this is something that can be developed through practise. Using objective measures of empathy before and after an SR may also be useful to determine if students’ empathy skills improve with these early interventions.

## Data Availability

All the response data for this study are displayed in Table 1. The other tables are correlations drawn from the data in Table 1. All data referred to in the report is included in the submitted manuscript.

## Abbreviations

SR: Schwartz Round
CE: Clinical Educator
GMC: General Medical Council
MBChB: Bachelors in Medicine
POCF: Point of Care Foundation
SPSS: Statistical Package for the Social Sciences
